# Vector competence of *Aedes aegypti*, *Culex tarsalis*, and *Culex quinquefasciatus* from California for Zika virus

**DOI:** 10.1371/journal.pntd.0006524

**Published:** 2018-06-21

**Authors:** Bradley J. Main, Jay Nicholson, Olivia C. Winokur, Cody Steiner, Kasen K. Riemersma, Jackson Stuart, Ryan Takeshita, Michelle Krasnec, Christopher M. Barker, Lark L. Coffey

**Affiliations:** 1 Department of Pathology, Microbiology, and Immunology, School of Veterinary Medicine, University of California Davis, Davis, California, United States of America; 2 Abt Associates Inc., Boulder, Colorado, United States of America; INDEPENDENT RESEARCHER, UNITED STATES

## Abstract

Zika virus (ZIKV) has emerged since 2013 as a significant global human health threat following outbreaks in the Pacific Islands and rapid spread throughout South and Central America. Severe congenital and neurological sequelae have been linked to ZIKV infections. Assessing the ability of common mosquito species to transmit ZIKV and characterizing variation in mosquito transmission of different ZIKV strains is important for estimating regional outbreak potential and for prioritizing local mosquito control strategies for *Aedes* and *Culex* species. In this study, we evaluated the laboratory vector competence of *Aedes aegypti*, *Culex quinquefasciatus*, and *Culex tarsalis* that originated in areas of California where ZIKV cases in travelers since 2015 were frequent. We compared infection, dissemination, and transmission rates by measuring ZIKV RNA levels in cohorts of mosquitoes that ingested blood meals from type I interferon-deficient mice infected with either a Puerto Rican ZIKV strain from 2015 (PR15), a Brazilian ZIKV strain from 2015 (BR15), or an ancestral Asian-lineage Malaysian ZIKV strain from 1966 (MA66). With PR15, *Cx*. *quinquefasciatus* was refractory to infection (0%, N = 42) and *Cx*. *tarsalis* was infected at 4% (N = 46). No ZIKV RNA was detected in saliva from either *Culex* species 14 or 21 days post feeding (dpf). In contrast, *Ae*. *aegypti* developed infection rates of 85% (PR15; N = 46), 90% (BR15; N = 20), and 81% (MA66; N = 85) 14 or 15 dpf. Although MA66-infected *Ae*. *aegypti* showed higher levels of ZIKV RNA in mosquito bodies and legs, transmission rates were not significantly different across virus strains (*P* = 0.13, Fisher’s exact test). To confirm infectivity and measure the transmitted ZIKV dose, we enumerated infectious ZIKV in *Ae*. *aegypti* saliva using Vero cell plaque assays. The expectorated plaque forming units PFU varied by viral strain: MA66-infected expectorated 13±4 PFU (mean±SE, N = 13) compared to 29±6 PFU for PR15-infected (N = 13) and 35±8 PFU for BR15-infected (N = 6; ANOVA, df = 2, F = 3.8, *P* = 0.035). These laboratory vector competence results support an emerging consensus that *Cx*. *tarsalis* and *Cx*. *quinquefasciatus* are not vectors of ZIKV. These results also indicate that *Ae*. *aegypti* from California are efficient laboratory vectors of ancestral and contemporary Asian lineage ZIKV.

## Author summary

Assessing the ability of common mosquito species to transmit Zika virus (ZIKV) and characterizing variation in mosquito transmission of different ZIKV strains is important for estimating regional outbreak potential and for prioritizing local mosquito control strategies for *Aedes* and *Culex* species. In this study, we evaluated the laboratory vector competence of *Aedes aegypti*, *Culex quinquefasciatus*, and *Culex tarsalis* that originated in areas of California where ZIKV cases in travelers since 2015 were frequent. We observed variation in infection loads between ZIKV strains in *Ae*. *aegypti*, but transmission rates were not different. In addition, there was a positive relationship between ZIKV RNA levels in infected mosquitoes ascertained from bodies and ZIKV RNA transmission rates. Our data add to the growing body of evidence supporting the role of *Aedes aegypti* as a ZIKV vector and refute *Cx*. *quinquefasciatus* and *Cx*. *tarsalis* as vectors.

## Introduction

Zika virus (ZIKV) is a mosquito-transmitted flavivirus that was first isolated in 1947 in the Zika forest of Uganda from a sentinel rhesus macaque [1]. Since its discovery, human ZIKV cases have been reported across Africa and Asia, but until 2007 the virus received little attention from researchers as it was thought to cause only mild disease. Following epidemics in Micronesia in 2007, French Polynesia in 2013, and Brazil in 2015 [2], ZIKV has now been confirmed as a cause of the neurological disease Guillain-Barre syndrome and congenital disorders, including microcephaly in infants [3]. Despite a dramatic decline in Brazilian cases since 2016, ZIKV remains a significant global human health threat [4], as other countries including Argentina, Bolivia, Peru, and Ecuador reported an increase in cases in 2017 [5].

Reducing mosquito vector populations is an effective way to mitigate mosquito-borne disease transmission [6]. Therefore, identifying ZIKV vector species is crucial for accurate risk assessments for mosquito transmission and to target vector control measures to mitigate ZIKV disease. Several *Aedes* species have been identified as competent vectors in laboratory studies, including the primary vector *Aedes* (*Ae*.) *aegypti* [7–21], *Ae*. *albopictus* [7,8,10,11,15,17,19,20,22,23], *Ae*. *notoscriptus* [10], *Ae*. *camptorhynchus* [10], *Ae*. *luteocephalus* [24], *Ae*. *vexans* [25], and *Ae*. *vittatus* [24]. *Culex* species generally do not become infected with ZIKV and are incapable of transmitting [7,9,10,12,14,17,23,26–29]. Exceptions include a study from Guadalajara, Mexico, where infectious ZIKV was detected in pooled mosquito tissue samples from field-collected *Cx*. *tarsalis*, *Cx*. *coronator*, and *Cx*. *quinquefasciatus* [30]. ZIKV RNA has also been detected in pooled field samples of *Cx*. *quinquefasciatus* from China [31]. Evidence for ZIKV transmission by *Culex* species is limited to *Cx*. *quinquefasciatus* and includes ZIKV RNA detected in saliva on Flinders Technology Associates (FTA) cards provided to a cohort of laboratory-infected mosquitoes from Brazil [32] and transmission to 1-day-old mice from mosquitoes from China, although inconsistently with other murine studies [33–35], no murine fatality was noted [36].

Previous studies demonstrate that ZIKV vector competence is more complex than simple mosquito species-level designations, and thus region-specific mosquito genotypes and multiple ZIKV strains must be evaluated to assess region-specific vector competence. For example, *Ae*. *aegypt*i from the Dominican Republic transmit ZIKV isolated from Cambodia in 2010 (FSS 13025) and Mexico in 2015 (MEX1-7) more effectively than *Ae*. *aegypti* from Salvador, Brazil [21]. Furthermore, ZIKV from Brazil in 2015 (BeH815744) has higher infectivity than a French Polynesian strain from 2013 (H/PF13) in *Ae*. *aegypti* from Singapore [8]. The source of virus also matters; fresh ZIKV was more infectious in comparative studies than freeze-thawed virus [12].

California (CA) vector control districts have been combating stable *Ae*. *aegypti* populations in the state since 2013 [37], including in many counties in Southern CA. In addition, between 2015 and March 2018, 640 travel-associated ZIKV infections were reported in CA [38], 137 (21% of cases in state) of which were in Los Angeles County where the *Ae*. *aegypti* used for vector competence experiments here were collected. Due to the presence of *Ae*. *aegypti* and numerous travel-associated ZIKV infections, there is a risk of the establishment of local mosquito-borne ZIKV transmission. Additionally, genetic variation between Central Valley and Southern CA *Ae*. *aegypti* populations has been observed [39], even between populations in neighboring cities, such as Fresno and Clovis [40]. These findings indicate that gene flow is limited between *Ae*. *aegypti* populations and leave open the possibility that important traits, such as vector competence, may also vary among *Ae*. *aegypti* throughout the state. To better assess local ZIKV transmission risk, we evaluated the laboratory vector competence of *Ae*. *aegypti* from Los Angeles, CA, for ZIKV isolates from Puerto Rico (2015), Brazil (2015), and Malaysia (1966). We also evaluated the laboratory vector competence of two highly abundant *Culex* species, *Cx*. *quinquefasciatus* from Orange County, CA, and *Cx*. *tarsalis* from Kern County, CA, with a Puerto Rico (2015) ZIKV strain.

## Materials and methods

### Sources of ZIKV strains, mosquitoes, and mice

Three Asian-lineage strains of ZIKV were used in our experiments. A 2015 Puerto Rican strain was isolated from human serum in 2015 (PR15, PRVABC59), passaged 4 times in Vero cells, and sequenced. The coding sequence for the complete genome of the passaged we used was identical to GenBank accession number KX601168. An Asian-lineage Malaysian ZIKV strain isolated from *Ae*. *aegypti* mosquitoes in 1966 (MA66, P6-740 [41]) that had been passaged in suckling mouse brains 6 times and once in Vero cells before it was received from the Centers for Disease Control was passaged once more in Vero cells. The complete coding genome sequence of our passage of MA66 was 100% identical to GenBank accession number KX601167.1. A Brazilian strain isolated from human serum in 2015 (BR15, SPH2015) was passaged 3 times in Vero cells and sequenced. The complete genome coding sequence of BR15 was identical to GenBank accession number KU321639. Strains MA66 and PR15 were obtained from Dr. Aaron Brault at the U.S. Centers for Disease Control and Prevention in Fort Collins, Colorado. Dr. Mike Busch at Blood Systems Research Institute, San Francisco, CA, provided the BR15 strain. All ZIKV strains and their source Vero cells were confirmed mycoplasma negative by PCR according to the manufacturer’s instructions (Agilent Mycoplasma Plus PCR Primer Kit, Santa Rosa, CA.)

The *Ae*. *aegypti* mosquitoes used in this study were field-collected as larvae in Los Angeles, CA, in 2016 and morphologically identified. The F6 generation was used for this study. Adult *Cx*. *quinquefasciatus* mosquitoes were field-collected as adults in Orange County, California in 2016 and morphologically identified. The F5 generation was used for this study. The *Cx*. *tarsalis* mosquitoes were field-collected in the Kern National Wildlife Refuge, Kern County, CA in 2002, morphologically identified, and have been maintained continuously in colony since.

Female interferon-deficient (IFN-α/βR−/−; C57BL/6) mice aged 4–8 weeks (B6.129S2-Ifnar1tm1Agt/Mmjax, The Jackson Laboratory, Sacramento, CA) were used for all experiments. Differences in ZIKV viremia levels and kinetics in male versus female mice have not been observed [33].

### ZIKV vector competence experiments

Mice were inoculated with 5 log_10_ Vero plaque forming units (PFU) of ZIKV via subcutaneous injection. ZIKV-infected mice were presented to mosquitoes 2 days post-inoculation, at peak viremia [33]. Mice were anesthetized prior to mosquito exposure with a ketamine (VETone Zetamine CIII, 75 mg/kg), xylazine (AnaSed, 10 mg/kg), and acepromazine (AceproJect, 1 mg/kg) solution administered intraperitoneally. The ZIKV viremia in each mouse was determined by Vero cell plaque assay from 30 μL of whole blood collected immediately prior to the mosquito feed. Viremic mice were presented for two cohorts of adult female mosquitoes 30–60 minutes on one of three arrangements depending on species: (1) 25 *Cx*. *tarsalis* in pint cartons (amazon.com), (2) 50 *Ae*. *aegypti* in pint cartons, or (3) >100 *Cx*. *quinquefasciatus* in a 1 ft^3^ mesh cage (BugDorm, MegaView Science, Taiwan). Engorged females were sorted from non-fed individuals by vacuum aspiration. Mosquito ages at the time of blood-feeding were 4–14 days post eclosion (dpe) for *Cx*. *tarsalis*, 14–21 dpe for *Cx*. *quinquefasciatus*, and 4–12 dpe for *Ae*. *aegypti*. *Cx*. *tarsalis* and *Ae*. *aegypti* were held at 26°C, 80% relative humidity, and 12:12 h light:dark cycle. *Cx*. *quinquefasciatus* were maintained at room temperature (22°C and 33% relative humidity) to ensure survival. All mosquitoes had constant access to 10% sucrose before and after blood-feeding, except during a 24-hour starvation period prior to presentation of the viremic mice. At days 14 and 21 post bloodfeed, mosquitoes were cold-anesthetized at -20°C for 5 minutes and then legs and wings were removed with forceps while immobilized on ice. Saliva was collected by inserting the proboscis into a capillary tube containing fetal bovine serum (FBS, GenClone) for 20 minutes. Individual bodies, legs+wings, and the saliva sample from each mosquito were stored separately in 2 mL tubes containing a 5 mm glass bead and 250 μL Dulbecco’s modified eagle medium (DMEM, Gibco) supplemented with 50μg/mL of penicillin/streptomycin and 20% FBS. All samples were stored at -80°C until further processing.

### ZIKV RNA extraction

Mosquito tissues and glass capillary tubes containing saliva samples were homogenized in DMEM by shaking for 2 minutes at 30 shakes/second using a Tissuelyser (Qiagen, Hilden, Germany). Viral RNA was extracted using the MaxMax Viral RNA Extraction Kit (ThermoFisher, Waltham, MA). A total of 50 μL of homogenate for mosquito tissue and 100 μL of saliva samples were extracted. All RNA extracts were eluted in 50 μL of elution buffer (Buffer EB, Qiagen) and stored at -80°C until further testing.

### ZIKV RT-qPCR

ZIKV RNA titers were determined for each body, legs+wings, and saliva sample using the Taqman Fast Virus One-Step Master Mix (ThermoFisher) reverse transcription RT-qPCR kit with a previously described ZIKV-specific assay (primers: ZIKV 1086, ZIKV 1162c, and ZIKV 1107-FAM; [42]). At least two technical replicates were performed for all samples. Samples with a mean cycle threshold (Ct) value of 38 or below were considered positive for ZIKV RNA. This limit of detection was determined from prior testing of serially diluted samples of known ZIKV RNA concentrations with the same extraction and RT-qPCR reagents and protocols and equipment [43].

### Infectivity of mosquito saliva

To estimate infectious ZIKV in expectorated *Ae*. *aegypti* saliva, viral titrations were performed on a random sample of RT-qPCR-positive saliva samples at the second or third thaw in Vero cell culture by plaque assay. In brief, cell monolayers were inoculated with 110 μL of undilute saliva from individual mosquitoes mixed with DMEM containing 2% (vol/vol) FBS, and 100 U/mL penicillin/streptomycin. After a one hour incubation period to allow for viral infection of cells, 0.8% agarose/DMEM was added to cover the cells. The plates were incubated at 37°C in 5% CO_2_ for 8 days. The cells were then fixed with 4% formaldehyde and stained with 0.05% crystal violet. Plaques were visualized as holes in the Vero cell monolayer and counted to determine PFU values. The limit of detection of the assay was 2.3 PFU where 110 μL of the total saliva sample (250 μL) was inoculated directly onto the cells. Since the volume of saliva was limited, each sample was tested in just 1 replicate.

### Infection definitions, cohort grouping, and statistical analyses

In this study, we calculated infection rates as the number of RT-qPCR positive individual bodies divided by the number of individuals that ingested blood and were tested, dissemination rates as the number of RT-qPCR positive pooled leg & wing sets from each individual divided by the number of individuals that ingested blood and were tested, and transmission rates as the number of RT-qPCR positive saliva samples divided by the total number of individuals that ingested blood and were tested. For *Ae*. *aegypti* and *Cx*. *tarsalis*, multiple cohorts of the same species fed on different mice infected with the same ZIKV strain with slight (≤1 log_10_) variations in viremias. Preliminary analysis across same-species cohorts that fed on different mice infected with the same ZIKV strain revealed no significant differences (Fisher’s exact test, *P*>0.05) in infection, dissemination and transmission rates. We therefore combined the data presented for each ZIKV strain for *Ae*. *aegypti* and *Cx*. *tarsalis*, while also reporting the magnitudes of viremia in all mice (Table 1). Comparisons of ZIKV RNA levels and PFU in saliva samples between ZIKV strains was performed using a one-way ANOVA with Tukey’s correction for pairwise comparisons (reported as *P*_adj_) and ZIKV RNA detection rates were compared using two-tailed Fisher’s exact tests (scipy.stats). Data were plotted using matplotlib (Python).

**Table 1:** ZIKV infection, dissemination, and transmission rates in California *Aedes* and *Culex* mosquitoes 14 and 21 days post ingestion of blood from viremic mice.

Mosquito Species	Source in California	Generation	ZIKV strain	Blood titer log_10_ PFU/mL	Infected (%)		Disseminated (%)		Transmitted (%)	
					Day 14	Day 21	Day 14	Day 21	Day 14	Day 21
***Cx*. *tarsalis***	**Kern County**	**Colony**	**PR15**	**5.7, 6.4, 5.4**	**2/46 (4)**	**6/20 (30)**	**2/46 (4)**	**1/20 (5)**	**0/46 (0)**	**0/20 (0)**
***Cx*. *quinquefasciatus***	**Los Angeles**	**F5**	**PR15**	**4.6**	**0/42 (0)**	**0/37 (0)**	**0/42 (0)**	**0/37 (0)**	**0/42 (0)**	**0/37 (0)**
***Ae*. *aegypti***	**Los Angeles**	**F6**	**MA66**	**4.3, 4.8**	**73/85 (86)**	**22/23 (96)**	**69/85 (79)**	**21/23 (91)**	**45/85 (53)**	**20/23 (87)**
***Ae*. *aegypti***	**Los Angeles**	**F6**	**PR15**	**5.7, 6.4, 5.4**	**39/46 (85)**	**22/23 (96)**	**36/46 (78)**	**18/23 (78)**	**30/46 (65)**	**17/23 (74)**
***Ae*. *aegypti***	**Los Angeles**	**F6**	**BR15**	**4.7**	18/20* (90)	**n.c.**	18/20*(90)	**n.c.**	15/20 *(75)	**n.c.**

Infection, dissemination, and transmission rates in mosquitoes that ingested ZIKV from viremic mice, determined by ZIKV RNA detection in bodies, legs+wings, and saliva, respectively. Denominators in rates represent all mosquitoes in cohorts. Multiple values in the mouse blood titer column show viremias for individual mice just before mosquitoes were presented to feed; cohorts of mosquitoes that fed on different mice within this range of viremias were combined since preliminary analysis of each cohort showed no differences in infection, dissemination and transmission rates (data not shown). n.c. indicates samples were not collected at that time point.

*Ae. *aegypti* that ingested BR15 were harvested 15 dpf.

### Ethics statement

All procedures involving mice were performed in accordance with IACUC protocol #19404 that was reviewed and approved by the UC Davis IACUC on June 29, 2017. The UC Davis IACUC adheres to the Office of Laboratory Animal Welfare Health Research Extension Act of 1985 (Public Law 99–158) as well as the United State Department of Agriculture’s Animal Welfare Act. UC Davis is accredited by the Association for Assessment and Accreditation of Laboratory Animal Care, International (AAALAC) and has an Animal Welfare Assurance (number A3433-01) on file with the Office of Laboratory Animal Welfare (OLAW).

## Results

### *Cx*. *tarsalis* and *Cx*. *quinquefasciatus* from California were incapable of transmitting Puerto Rican ZIKV in laboratory vector competence experiments

*Cx*. *tarsalis* and *Cx*. *quinquefasciatus* mosquitoes were tested 14 or 21 days after ingesting blood from ZIKV-infected interferon receptor deficient mice. Two *Cx*. *tarsalis* bodies out of the 46 individuals tested (4%) had low levels of ZIKV RNA at 14 dpf (Ct < 38; 48 ZIKV genomes/body). Both infected individuals also had detectable ZIKV in their legs and wings, indicating disseminated infections. Neither of the ZIKV-infected *Cx*. *tarsalis* contained detectable ZIKV RNA in their saliva samples (Table 1). The *Cx*. *tarsalis* infection rate significantly increased from 4% to 30% (2/46 to 6/20, *P*<0.01 Fisher exact test) from 14 to 21 dpf. Among the 6 infected *Cx*. *tarsalis* at 21 dpf, ZIKV RNA was detected in 1 leg and wing sample but, consistent with a lack of transmission 14 dpf, no ZIKV RNA was detected in the saliva (Table 1). We did not detect ZIKV RNA in any *Cx*. *quinquefasciatus* mosquito tissues 14 (N = 42) or 21 dpf (N = 37; Table 1).

### *Ae*. *aegypti* from Los Angeles, CA, were highly competent laboratory ZIKV vectors

At 14 dpf, ZIKV infection, dissemination, and transmission rates measured by the presence of ZIKV RNA in *Ae*. *aegypti* that ingested MA66 were 86%, 79%, and 53%, respectively (Table 1). For *Ae*. *aegypti* that ingested ZIKV PR15, the infection, dissemination, and transmission rates on 14 dpf were 85%, 78%, and 65%, respectively (Table 1). ZIKV BR15-exposed individuals harvested 15 dpf had infection, dissemination, and transmission rates of 90%, 90%, and 75%, respectively (Table 1). ZIKV RNA infection, dissemination, and transmission rates in *Ae*. *aegypti* that ingested MA66 or PR15 at 21 dpf were equal or higher than 14 dpf rates. The transmission rate between 14 and 21 dpf increased significantly in *Ae*. *aegypti* infected with MA66 (53% vs. 87%, *P*<0.01, Fisher’s exact), but not PR15 (65% vs. 74%, *P* = 0.59, Fisher’s exact; Table 1). Transmission rates were not significantly different across viruses (*P* = 0.13, Fisher’s exact).

**Fig 1:**
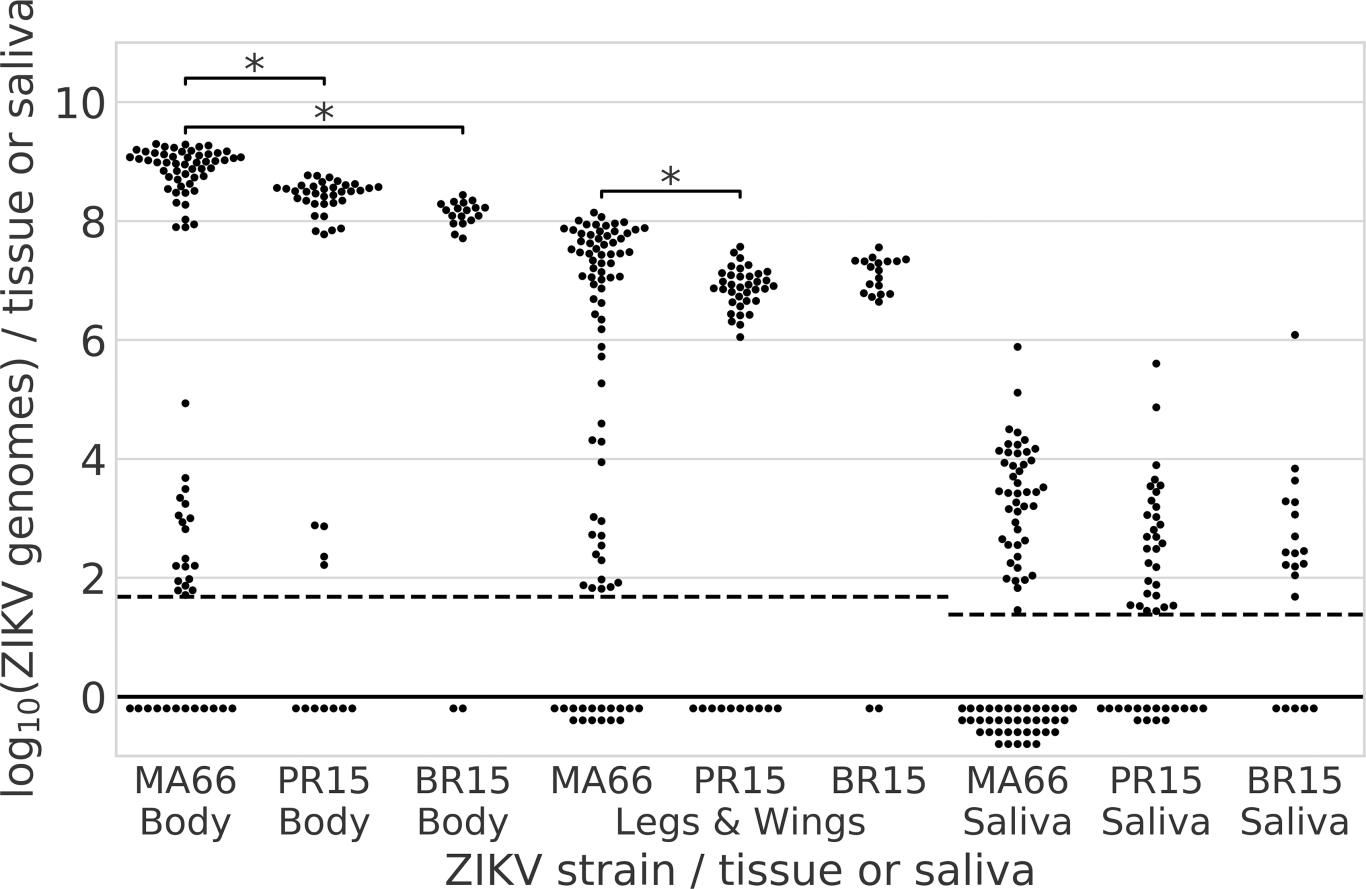
Infecting (bodies), disseminating (legs+wings) and transmitted (saliva) ZIKV RNA levels 14 or 15 days after *Ae*. *aegypti* orally ingested one of three Asian lineage ZIKV strains. Each dot represents the mean log_10_ ZIKV genome copies per tissue or saliva sample from an individual *Ae*. *aegypti*. *Ae*. *aegypti* from Los Angeles, California, USA, were fed on viremic mice infected with ZIKV from Malaysia 1966 (MA66), Puerto Rico 2015 (PR15) or Brazil 2015 (BR15). Mosquitoes exposed to BR15 were assayed 15 dpf, MA66 and PR15 cohorts were assayed 14 dpf. Each dot represents the mean of two or more RT-qPCR technical replicates. The dashed lines represent the limits of detection (LOD). Dots below dashed line represent tested samples with an undetectable Ct or a Ct value of >38. The LOD for saliva was lower than for tissues because RNA was extracted from a higher proportion of the saliva sample volume. Asterisks show significant differences in means across groups of the same tissue type, *P*<0.01, ANOVA, Tukey post-hoc test. No asterisk indicates no significant difference across groups.

The mean ZIKV RNA level (8.9 log_10_) in MA66-infected bodies was significantly higher than the mean for BR15 (8.2 log_10_, ANOVA, degrees of freedom (df) = 2, F-statistic (F) = 16.3, *P*_adj_<0.01) and PR15-infected individuals (8.4 log_10_, ANOVA, df = 2, F = 16.3, *P*_adj_<0.01; Fig 1). The mean ZIKV RNA level in MA66-infected leg+wing tissue (7.5 log_10_) was also significantly higher than PR15-infected leg+wing samples (7.0 log_10_, ANOVA, df = 2, F = 8.4, *P*_adj_<0.01). Higher ZIKV RNA levels in MA66-infected *Ae*. *aegypti* likely do not reflect the dose ingested, where flavivirus infections of mosquitoes typically show a strong dose response, since viremias in both ZIKV MA66-infected mice were lower than those for PR15. ZIKV RNA levels in saliva were not significantly different among strains (ANOVA, df = 2, F = 0.96, *P* = 0.39).

**Fig 2:**
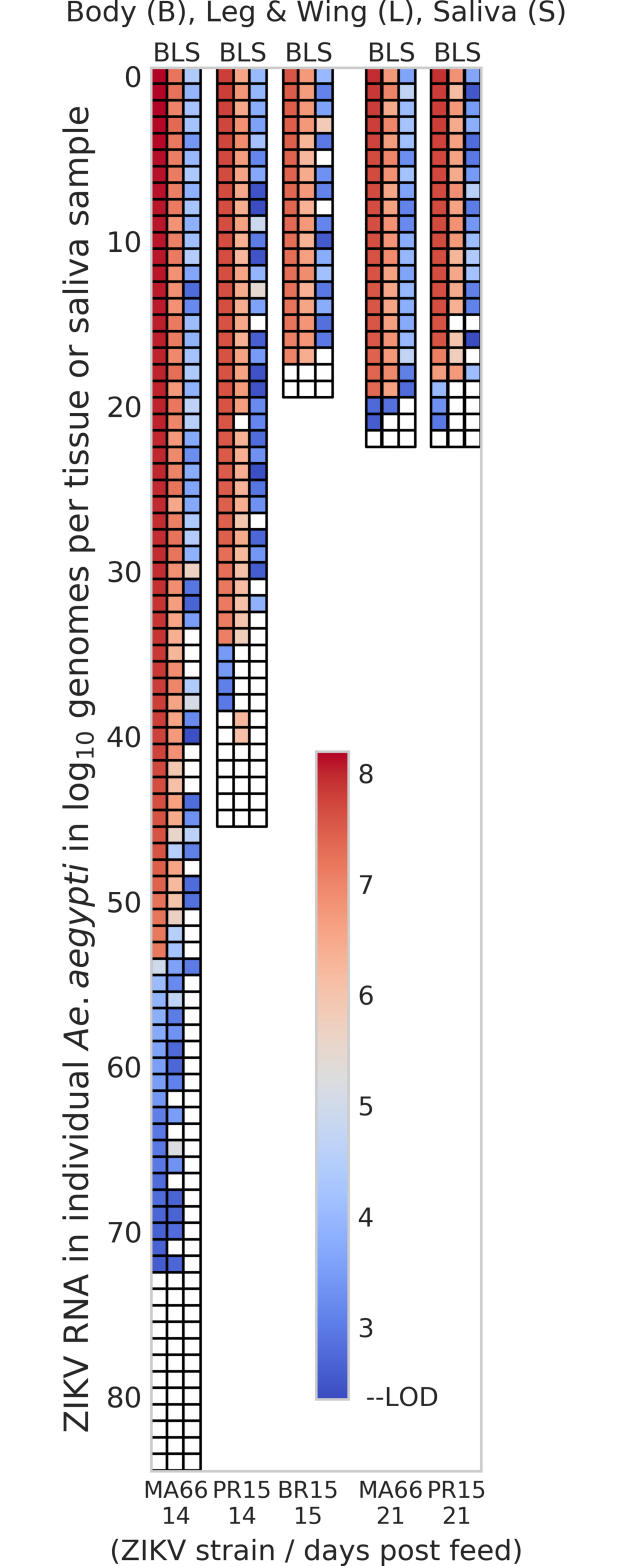
Individual mosquito ZIKV RNA levels in body, legs+wings, and saliva of *Aedes aegypti* from Los Angeles, CA, USA. *Ae*. *aegypti* from Los Angeles ingested blood from viremic ZIKV-infected interferon receptor deficient mice that had been inoculated with different ZIKV strains. Each colored box represents an individual mosquito sample showing the magnitude of ZIKV RNA detected in each body (B), legs+wings (LW), and saliva (S). The red to blue color scale shows high (red) to low (blue) ZIKV RNA levels. Samples with no detectable ZIKV RNA are colored white. ZIKV BR15-infected mosquitoes were not tested 21 dpf.

A bimodal distribution of ZIKV RNA levels was observed across cohorts of ZIKV PR15- or MA66-infected bodies, with high (>6 log_10_ genomes/body) and low (<6 log_10_ genomes/body) clusters of individuals (Fig 1). MA66-infected *Ae*. *aegypti* that were highly infected (>6 log_10_ genomes/body) had higher transmission rates (81%, N = 54) compared to low titer (<6 log_10_ genomes/body) individuals (5%, N = 19; *P*<0.0001, Fisher’s exact). We also examined the relationship between infection, dissemination and transmission at an individual mosquito level for *Ae*. *aegypti* (Fig 2). Most *Ae*. *aegypti* that became infected developed disseminated infections. Individuals with higher (red/pink in figure) ZIKV RNA levels in legs+wings were more likely to transmit ZIKV RNA than mosquitoes with low (blue in figure) RNA levels in legs+wings. None of the PR15-infected *Ae*. *aegypti* with <6 log_10_ genomes/body transmitted ZIKV RNA (N = 4).

**Fig 3:**
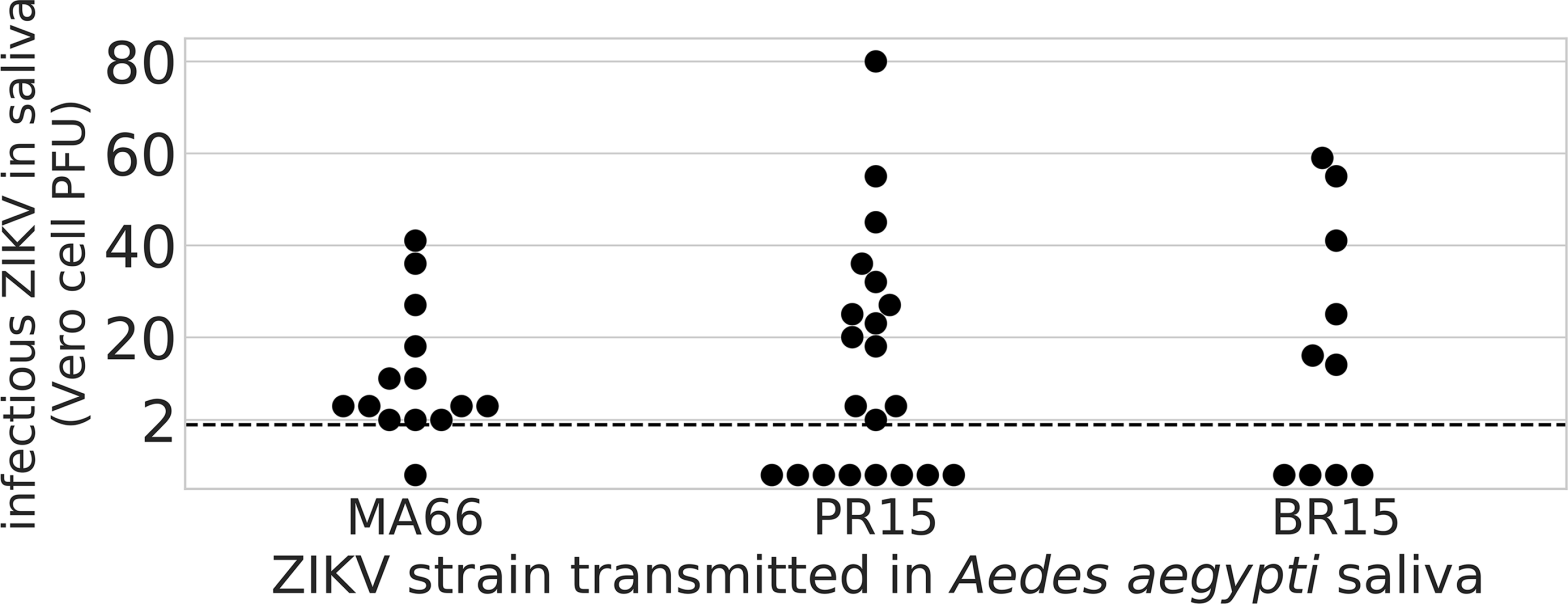
Infectious ZIKV levels in expectorated *Ae*. *aegypti* saliva. Saliva from *Ae*. *aegypti* mosquitoes from Los Angeles, CA, USA, infected with either MA66, PR15, or BR15 ZIKV was collected in capillary tubes 14 (MA66 and PR15) or 15 (BR15) dpf. Vero cell plaque assays were performed on RT-qPCR positive saliva expectorants to quantify infectious viruses. The limit of detection (LOD) of the assay was 2.3 PFU and is shown as a dashed line. Dots below the dashed line represent saliva samples with no detectable plaques.

To confirm infectivity and measure the transmitted ZIKV dose, plaque assays were performed on *Ae*. *aegypti* saliva collected 14 or 15 dpf to enumerate infectious ZIKV in Vero cell plaque forming units (PFU). Out of 45 RTq-PCR positive saliva samples that were tested by plaque assay, 32 (71%) yielded at least 1 detectable plaque. The expectorated PFU varied by viral strain: the MA66-infected individuals transmitted 13±4 PFU (mean±SE, N = 13) compared to 29±6 for PR15 (N = 13) and 35±8 for BR15 (N = 6; ANOVA, df = 2, F = 3.8, *P* = 0.035; Fig 3).

## Discussion

Understanding the mosquito species that vector ZIKV is important for estimating regional outbreak potential and for informing local mosquito control strategies, especially since *Aedes* and *Culex* species differ in life history traits and host-seeking behaviors that could impact control efforts. For example, oviposition traps bias towards *Ae*. *aegypti* that lay in artificial containers [44] while *Culex* typically prefer natural pools [45]. For *Cx*. *tarsalis*, we detected an overall ZIKV infection rate of 12% (8/66) in mosquitoes tested 14 and 21 dpf. Disseminated infections in *Cx*. *tarsalis* were detected at <5% on both 14 and 21 dpf, with high Ct values indicating low ZIKV RNA levels. We postulate that the disseminated infections detected in *Cx*. *tarsalis* may reflect false positives given that mosquitoes with true disseminated infections typically achieve very high viral RNA titers due to prolonged infection of multiple tissues. The absence of detectable ZIKV RNA in saliva at 14 or 21 dpf is evidence that *Cx*. *tarsalis* from CA is not capable of transmitting ZIKV in laboratory experiments. Furthermore, *Cx*. *tarsalis* feeds less often on human hosts compared to the highly anthropophilic *Ae*. *aegypti* [45–47], making human-mosquito-human ZIKV transmission by *Cx*. *tarsalis* unlikely. We also found no evidence for ZIKV infection of *Cx*. *quinquefasciatus* from California, with no ZIKV RNA detected in bodies, legs/wings or saliva from nearly 80 individuals. This is the first data showing ZIKV vector competence for California mosquitoes, and it supports results from many other studies which demonstrate that *Cx*. *quinquefasciatus* is not a competent laboratory vector of ZIKV. By contrast, *Ae*. *aegypti* mosquitoes exhibited infection rates of 85–90% and transmission rates of 53–80% at 14 dpf. The transmitted dose of infectious ZIKV by Californian *Ae*. *aegypti* is consistent with the range of doses observed in similar studies with Brazilian *Ae*. *aegypti* [48,49].

*Ae*. *aegypti* that ingested ZIKV MA66 in our laboratory vector competence studies developed higher ZIKV RNA levels than PR15- or BR15-infected mosquitoes. This pattern contrasted with the lower transmission rate and lower expectorated PFU of MA66-infected *Ae*. *aegypti* at 14 dpf.

A possible explanation for the lower transmissibility of MA66 at 14 dpf is that it lacks a A188V mutation in the NS1 gene that both PR15 and BR15 possess, which has been linked to higher infectivity (where infectivity can influence transmissibility) in mosquitoes when interferon-deficient mice are used for blood-feeding [7]. ZIKV strains from recent American outbreaks have also been shown to exhibit higher infection and transmission rates than historic Asian-lineage strains [8]. Additional vector competence studies involving region-specific *Ae*. *aegypti* and *Ae*. *albopictus* mosquito populations with sequenced genomes and multiple distinct ZIKV isolates will improve our understanding of the both mosquito and virus genetics involved in ZIKV vector competence, which could inform our ability to accurately estimate regional outbreak potential.

Among ZIKV MA66-infected *Ae*. *aegypti*, we observed that mosquitoes with low RNA copy numbers in bodies were less likely to transmit than those with infections that exceeded 6 log_10_ genomes per body. This pattern is consistent with the presence of a midgut barrier to infection [50]. In that case, the mosquitoes with low body RNA levels represent infections that have not escaped the midgut while mosquitoes with high body RNA levels correspond to individuals with ZIKV that has disseminated to secondary amplification tissues.

This laboratory vector competence study confirmed that *Ae*. *aegypti* from Los Angeles, California, USA, can transmit Asian lineage ZIKV and that *Cx*. *tarsalis* and *Cx*. *quinquefasciatus* are inefficient ZIKV vectors. Given that *Culex* mosquitoes are poor ZIKV vectors and seek primarily non-human hosts, they are unlikely to facilitate a ZIKV outbreak. Thus, vector control efforts targeting ZIKV should remain focused on reducing urban *Aedes* populations.

## References

[1] Dick GWA, Kitchen SF, Haddow AJ. Zika Virus (I). Isolations and serological specificity. Trans R Soc Trop Med Hyg. Oxford University Press; 1952;46: 509–520. 12995440 10.1016/0035-9203(52)90042-4

[2] Kindhauser MK, Allen T, Frank V, Santhana RS, Dye C. Zika: the origin and spread of a mosquito-borne virus. Bull World Health Organ. who.int; 2016;94: 675–686C. doi: 10.2471/BLT.16.171082 27708473 10.2471/BLT.16.171082 PMC5034643

[3] Krauer F, Riesen M, Reveiz L, Oladapo OT, Martínez-Vega R, Porgo TV, et al Zika Virus Infection as a Cause of Congenital Brain Abnormalities and Guillain–Barré Syndrome: Systematic Review. PLoS Med. Public Library of Science; 2017;14: e1002203 doi: 10.1371/journal.pmed.1002203 28045901 10.1371/journal.pmed.1002203 PMC5207634

[4] WHO | Zika situation report. World Health Organization; 2017; Available: http://who.int/entity/emergencies/zika-virus/situation-report/10-march-2017/en/index.html

[5] Sanchez JD. PAHO WHO | Regional Zika Epidemiological Update (Americas) July 26, 2017. In: Pan American Health Organization / World Health Organization [Internet]. 15 Aug 2017 [cited 23 Aug 2017]. Available: http://www.paho.org/hq/index.php?option=com_content&id=11599&Itemid=41691

[6] Curtis CF, Davies CR. Present use of pesticides for vector and allergen control and future requirements. Med Vet Entomol. Blackwell Science, Ltd; 2001;15: 231–235. 11583439 10.1046/j.1365-2915.2001.00293.x

[7] Liu Y, Liu J, Du S, Shan C, Nie K, Zhang R, et al Evolutionary enhancement of Zika virus infectivity in *Aedes aegypti* mosquitoes. Nature. nature.com; 2017;545: 482–486. doi: 10.1038/nature22365 28514450 10.1038/nature22365 PMC5885636

[8] Pompon J, Morales-Vargas R, Manuel M, Huat Tan C, Vial T, Hao Tan J, et al A Zika virus from America is more efficiently transmitted than an Asian virus by *Aedes aegypti* mosquitoes from Asia. Sci Rep. 2017;7: 1215 doi: 10.1038/s41598-017-01282-6 28450714 10.1038/s41598-017-01282-6 PMC5430906

[9] Fernandes RS, Campos SS, Ferreira-de-Brito A, de Miranda RM, da Silva KAB, de Castro MG, et al *Culex quinquefasciatus* from Rio de Janeiro Is Not Competent to Transmit the Local Zika Virus. PLoS Negl Trop Dis. Public Library of Science; 2016;10: e0004993 doi: 10.1371/journal.pntd.0004993 27598421 10.1371/journal.pntd.0004993 PMC5012671

[10] Duchemin J-B, Mee PT, Lynch SE, Vedururu R, Trinidad L, Paradkar P. Zika vector transmission risk in temperate Australia: a vector competence study. Virol J. BioMed Central; 2017;14: 108 doi: 10.1186/s12985-017-0772-y 28599659 10.1186/s12985-017-0772-y PMC5466793

[11] Ciota AT, Bialosuknia SM, Zink SD, Brecher M, Ehrbar DJ, Morrissette MN, et al Effects of Zika Virus Strain and *Aedes* Mosquito Species on Vector Competence. Emerg Infect Dis. 2017;23: 1110–1117. doi: 10.3201/eid2307.161633 28430564 10.3201/eid2307.161633 PMC5512477

[12] Weger-Lucarelli J, Rückert C, Chotiwan N, Nguyen C, Garcia Luna SM, Fauver JR, et al Vector Competence of American Mosquitoes for Three Strains of Zika Virus. PLoS Negl Trop Dis. journals.plos.org; 2016;10: e0005101 doi: 10.1371/journal.pntd.0005101 27783679 10.1371/journal.pntd.0005101 PMC5081193

[13] Costa-da-Silva AL, Ioshino RS, Araújo HRC de, Kojin BB, Zanotto PM de A, Oliveira DBL, et al Laboratory strains of *Aedes aegypti* are competent to Brazilian Zika virus. PLoS One. 2017;12: e0171951 doi: 10.1371/journal.pone.0171951 28187183 10.1371/journal.pone.0171951 PMC5302382

[14] Hall-Mendelin S, Pyke AT, Moore PR, Mackay IM, McMahon JL, Ritchie SA, et al Assessment of Local Mosquito Species Incriminates *Aedes aegypti* as the Potential Vector of Zika Virus in Australia. PLoS Negl Trop Dis. Public Library of Science; 2016;10: e0004959 doi: 10.1371/journal.pntd.0004959 27643685 10.1371/journal.pntd.0004959 PMC5028067

[15] Aliota MT, Peinado SA, Velez ID, Osorio JE. The wMel strain of Wolbachia Reduces Transmission of Zika virus by *Aedes aegypti*. Sci Rep. The Author(s); 2016;6: 28792 doi: 10.1038/srep28792 27364935 10.1038/srep28792 PMC4929456

[16] Richard V, Paoaafaite T, Cao-Lormeau V-M. Vector Competence of French Polynesian *Aedes aegypti* and *Aedes polynesiensis* for Zika Virus. PLoS Negl Trop Dis. 2016;10: e0005024 doi: 10.1371/journal.pntd.0005024 27654962 10.1371/journal.pntd.0005024 PMC5031459

[17] Heitmann A, Jansen S, Lühken R, Leggewie M, Badusche M, Pluskota B, et al Experimental transmission of Zika virus by mosquitoes from central Europe. Euro Surveill. 2017;22 doi: 10.2807/1560-7917.ES.2017.22.2.30437 28106528 10.2807/1560-7917.ES.2017.22.2.30437 PMC5404485

[18] Boccolini D, Toma L, Di Luca M, Severini F, Romi R, Remoli ME, et al Experimental investigation of the susceptibility of Italian *Culex pipiens* mosquitoes to Zika virus infection. Euro Surveill. ncbi.nlm.nih.gov; 2016;21 doi: 10.2807/1560-7917.ES.2016.21.35.30328 27605056 10.2807/1560-7917.ES.2016.21.35.30328 PMC5015456

[19] Chouin-Carneiro T, Vega-Rua A, Vazeille M, Yebakima A, Girod R, Goindin D, et al Differential Susceptibilities of *Aedes aegypti* and *Aedes albopictus* from the Americas to Zika Virus. PLoS Negl Trop Dis. journals.plos.org; 2016;10: e0004543 doi: 10.1371/journal.pntd.0004543 26938868 10.1371/journal.pntd.0004543 PMC4777396

[20] Di Luca M, Severini F, Toma L, Boccolini D, Romi R, Remoli ME, et al Experimental studies of susceptibility of Italian *Aedes albopictus* to Zika virus. Euro Surveill. e-sciencecentral.org; 2016;21 doi: 10.2807/1560-7917.ES.2016.21.18.30223 27171034 10.2807/1560-7917.ES.2016.21.18.30223

[21] Roundy CM, Azar SR, Rossi SL, Huang JH, Leal G, Yun R, et al Variation in *Aedes aegypti* Mosquito Competence for Zika Virus Transmission. Emerg Infect Dis. 2017;23: 625–632. doi: 10.3201/eid2304.161484 28287375 10.3201/eid2304.161484 PMC5367433

[22] Wong P-SJ, Li M-ZI, Chong C-S, Ng L-C, Tan C-H. *Aedes (Stegomyia) albopictus* (Skuse): A Potential Vector of Zika Virus in Singapore. PLoS Negl Trop Dis. Public Library of Science; 2013;7: e2348 doi: 10.1371/journal.pntd.0002348 23936579 10.1371/journal.pntd.0002348 PMC3731215

[23] Azar SR, Roundy CM, Rossi SL, Huang JH, Leal G, Yun R, et al Differential Vector Competency of *Aedes albopictus* Populations from the Americas for Zika Virus. Am J Trop Med Hyg. The American Society of Tropical Medicine and Hygiene; 2017;97: 330–339. doi: 10.4269/ajtmh.16-0969 28829735 10.4269/ajtmh.16-0969 PMC5544086

[24] Diagne CT, Diallo D, Faye O, Ba Y, Faye O, Gaye A, et al Potential of selected Senegalese *Aedes* spp. mosquitoes (Diptera: Culicidae) to transmit Zika virus. BMC Infect Dis. 2015;15: 492 doi: 10.1186/s12879-015-1231-2 26527535 10.1186/s12879-015-1231-2 PMC4629289

[25] Gendernalik A, Weger-Lucarelli J, Garcia Luna SM, Fauver JR, Rückert C, Murrieta RA, et al American *Aedes vexans* Mosquitoes are Competent Vectors of Zika Virus. Am J Trop Med Hyg. ASTMH; 2017;96: 1338–1340. doi: 10.4269/ajtmh.16-0963 28719283 10.4269/ajtmh.16-0963 PMC5462567

[26] Amraoui F, Atyame-Nten C, Vega-Rúa A, Lourenço-de-Oliveira R, Vazeille M, Failloux AB. *Culex* mosquitoes are experimentally unable to transmit Zika virus. Euro Surveill. 2016;21 doi: 10.2807/1560-7917.ES.2016.21.35.30333 27605159 10.2807/1560-7917.ES.2016.21.35.30333 PMC5015461

[27] Dodson BL, Rasgon JL. Vector competence of *Anopheles* and *Culex* mosquitoes for Zika virus. PeerJ. 2017;5: e3096 doi: 10.7717/peerj.3096 28316896 10.7717/peerj.3096 PMC5354110

[28] Kenney JL, Romo H, Duggal NK, Tzeng W-P, Burkhalter KL, Brault AC, et al Transmission Incompetence of *Culex quinquefasciatus* and *Culex pipiens pipiens* from North America for Zika Virus. Am J Trop Med Hyg. 2017;96: 1235–1240. doi: 10.4269/ajtmh.16-0865 28500817 10.4269/ajtmh.16-0865 PMC5417222

[29] Huang Y-JS, Ayers VB, Lyons AC, Unlu I, Alto BW, Cohnstaedt LW, et al *Culex* Species Mosquitoes and Zika Virus. Vector Borne Zoonotic Dis. 2016;16: 673–676. doi: 10.1089/vbz.2016.2058 27556838 10.1089/vbz.2016.2058

[30] Elizondo-Quiroga D, Medina-Sánchez A, Sánchez-González JM, Eckert KA, Villalobos-Sánchez E, Navarro-Zúñiga AR, et al Zika Virus in Salivary Glands of Five Different Species of Wild-Caught Mosquitoes from Mexico. Sci Rep. 2018;8: 809 doi: 10.1038/s41598-017-18682-3 29339746 10.1038/s41598-017-18682-3 PMC5770420

[31] Fu S, Song S, Liu H, Li Y, Li X, Gao X, et al ZIKA virus isolated from mosquitoes: a field and laboratory investigation in China, 2016. Sci China Life Sci. 2017; doi: 10.1007/s11427-017-9196-8 29058107 10.1007/s11427-017-9196-8

[32] Guedes DR, Paiva MH, Donato MM, Barbosa PP, Krokovsky L, Rocha SWDS, et al Zika virus replication in the mosquito *Culex quinquefasciatus* in Brazil. Emerg Microbes Infect. 2017;6: e69 doi: 10.1038/emi.2017.59 28790458 10.1038/emi.2017.59 PMC5583667

[33] Rossi SL, Tesh RB, Azar SR, Muruato AE, Hanley KA, Auguste AJ, et al Characterization of a Novel Murine Model to Study Zika Virus. Am J Trop Med Hyg. 2016;94: 1362–1369. doi: 10.4269/ajtmh.16-0111 27022155 10.4269/ajtmh.16-0111 PMC4889758

[34] Lazear HM, Govero J, Smith AM, Platt DJ, Fernandez E, Miner JJ, et al A Mouse Model of Zika Virus Pathogenesis. Cell Host Microbe. 2016;19: 720–730. doi: 10.1016/j.chom.2016.03.010 27066744 10.1016/j.chom.2016.03.010 PMC4866885

[35] Miner JJ, Sene A, Richner JM, Smith AM, Santeford A, Ban N, et al Zika Virus Infection in Mice Causes Panuveitis with Shedding of Virus in Tears. Cell Rep. Elsevier; 2016;16: 3208–3218. doi: 10.1016/j.celrep.2016.08.079 27612415 10.1016/j.celrep.2016.08.079 PMC5040391

[36] Guo X-X, Li C-X, Deng Y-Q, Xing D, Liu Q-M, Wu Q, et al *Culex pipiens quinquefasciatus*: a potential vector to transmit Zika virus. Emerg Microbes Infect. 2016;5: e102 doi: 10.1038/emi.2016.102 27599470 10.1038/emi.2016.102 PMC5113053

[37] Gloria-Soria A, Brown JE, Kramer V, Hardstone Yoshimizu M, Powell JR. Origin of the dengue fever mosquito, *Aedes aegypti*, in California. PLoS Negl Trop Dis. 2014;8: e3029 doi: 10.1371/journal.pntd.0003029 25077804 10.1371/journal.pntd.0003029 PMC4117443

[38] CDPH [Internet]. 5 Jan 2018. Available: https://www.cdph.ca.gov/Programs/CID/DCDC/Pages/Zika.aspx

[39] Pless E, Gloria-Soria A, Evans BR, Kramer V, Bolling BG, Tabachnick WJ, et al Multiple introductions of the dengue vector, *Aedes aegypti*, into California. PLoS Negl Trop Dis. Public Library of Science; 2017;11: e0005718 doi: 10.1371/journal.pntd.0005718 28796789 10.1371/journal.pntd.0005718 PMC5552028

[40] Lee Y, Collier TC, Conner WR, Hanemaaijer MJ. Spatial and temporal distribution of genome divergence among California populations of Aedes aegypti. bioRxiv. biorxiv.org; 2017; Available: http://www.biorxiv.org/content/early/2017/07/20/166629.abstract

[41] Haddow AD, Schuh AJ, Yasuda CY, Kasper MR, Heang V, Huy R, et al Genetic characterization of Zika virus strains: geographic expansion of the Asian lineage. PLoS Negl Trop Dis. journals.plos.org; 2012;6: e1477 doi: 10.1371/journal.pntd.0001477 22389730 10.1371/journal.pntd.0001477 PMC3289602

[42] Lanciotti RS, Kosoy OL, Laven JJ, Velez JO, Lambert AJ, Johnson AJ, et al Genetic and serologic properties of Zika virus associated with an epidemic, Yap State, Micronesia, 2007. Emerg Infect Dis. 2008;14: 1232–1239. doi: 10.3201/eid1408.080287 18680646 10.3201/eid1408.080287 PMC2600394

[43] Stone M, Lanteri MC, Bakkour S, Deng X, Galel SA, Linnen JM, et al Relative analytical sensitivity of donor nucleic acid amplification technology screening and diagnostic real-time polymerase chain reaction assays for detection of Zika virus RNA. Transfusion. 2017;57: 734–747. doi: 10.1111/trf.14031 28194799 10.1111/trf.14031

[44] Ritchie SA, Long S, Hart A, Webb CE, Russell RC. An adulticidal sticky ovitrap for sampling container-breeding mosquitoes. J Am Mosq Control Assoc. 2003;19: 235–242. 14524545

[45] Reiter P, Others. A portable battery-powered trap for collecting gravid *Culex* mosquitoes. Mosq News. 1983;43: 496–498.

[46] Thiemann TC, Wheeler SS, Barker CM, Reisen WK. Mosquito host selection varies seasonally with host availability and mosquito density. PLoS Negl Trop Dis. 2011;5: e1452 doi: 10.1371/journal.pntd.0001452 22206038 10.1371/journal.pntd.0001452 PMC3243726

[47] Takken W, Verhulst NO. Host preferences of blood-feeding mosquitoes. Annu Rev Entomol. Annual Reviews; 2013;58: 433–453. doi: 10.1146/annurev-ento-120811-153618 23020619 10.1146/annurev-ento-120811-153618

[48] Roundy Christopher M., Azar Sasha R., Rossi Shannan L., Huang Jing H., Leal Grace, Yun Ruimei, et al Variation in *Aedes aegypti* Mosquito Competence for Zika Virus Transmission. Emerging Infectious Diseases. 2017;23: 625–632. doi: 10.3201/eid2304.161484 28287375 10.3201/eid2304.161484 PMC5367433

[49] Fernandes RS, Campos SS, Ribeiro PS, Raphael LM, Bonaldo MC, Lourenço-de-Oliveira R. *Culex quinquefasciatus* from areas with the highest incidence of microcephaly associated with Zika virus infections in the Northeast Region of Brazil are refractory to the virus. Mem Inst Oswaldo Cruz. 2017;112: 577–579. doi: 10.1590/0074-02760170145 28767975 10.1590/0074-02760170145 PMC5530542

[50] Turell MJ, Gargan TP 2nd, Bailey CL. Replication and dissemination of Rift Valley fever virus in *Culex pipiens*. Am J Trop Med Hyg. 1984;33: 176–181. 6696176 10.4269/ajtmh.1984.33.176

